# Active cooling of twisted coiled actuators via fabric air channels

**DOI:** 10.3389/fresc.2022.1016355

**Published:** 2022-11-30

**Authors:** Alex Lizotte, Ana Luisa Trejos

**Affiliations:** ^1^ WearME Lab, Department of Electrical and Computer Engineering, Western University, London, ON, Canada; ^2^ School of Biomedical Engineering, Western University, London, ON, Canada

**Keywords:** fabric channel, twisted coiled actuators, active cooling, soft robotics, artificial muscles

## Abstract

Twisted coiled actuators (TCAs) are promising artificial muscles for wearable soft robotic devices due to their biomimetic properties, inherent compliance, and slim profile. These artificial muscles are created by super-coiling nylon thread and are thermally actuated. Unfortunately, their slow natural cooling rate limits their feasibility when used in wearable devices for upper limb rehabilitation. Thus, a novel cooling apparatus for TCAs was specifically designed for implementation in soft robotic devices. The cooling apparatus consists of a flexible fabric channel made from nylon pack cloth. The fabric channel is lightweight and could be sewn onto other garments for assembly into a soft robotic device. The TCA is placed in the channel, and a miniature air pump is used to blow air through it to enable active cooling. The impact of channel size on TCA performance was assessed by testing nine fabric channel sizes—combinations of three widths (6, 8, and 10 mm) and three heights (4, 6, and 8 mm). Overall, the performance of the TCA improved as the channel dimensions increased, with the combination of a 10 mm width and an 8 mm height resulting in the best balance between cooling time, heating time, and stroke. This channel was utilized in a follow-up experiment to determine the impact of the cooling apparatus on TCA performance. In comparison to passive cooling without a channel, the channel and miniature air pump reduced the TCA cooling time by 42% (21.71±1.24 s to 12.54±2.31 s, p<0.001). Unfortunately, there was also a 9% increase in the heating time (3.46±0.71 s to 3.76±0.71 s, p<0.001) and a 28% decrease in the stroke (5.40±0.44 mm to 3.89±0.77 mm, p<0.001). This work demonstrates that fabric cooling channels are a viable option for cooling thermally actuated artificial muscles within a soft wearable device. Future work can continue to improve the channel design by experimenting with other configurations and materials.

## 1. Introduction

Each year, approximately 62,000 Canadians suffer a stroke (1). It is recommended that stroke patients receive at minimum 45 min of rehabilitation therapy 5 days per week, which creates high demands on physiotherapists (2). High quantities of rehabilitation are important, as it has been shown that increasing the amount of time spent completing physiotherapy exercises improves the ability of the patient to complete activities of daily living independently (3). One method of increasing access to rehabilitation is to create inexpensive, portable, wearable robotic systems that individuals can use at home and outside of the clinic to complete physiotherapy exercises. Review papers have found that robotic therapy can provide the same benefits as conventional physiotherapy, and that upper limb functions improved when robotic therapy was completed in addition to conventional therapy, especially with chronic stroke patients (4,5). Robotic therapy has many advantages such as progress tracking, real-time feedback, and reducing therapist workloads.

These robotic devices are not common due to their high cost and limited portability, which stems in part from using electric motors as the primary actuator (6–8). One method to reduce the cost and increase the portability of wearable robotic devices is to use alternative actuators, such as artificial muscles. Artificial muscles have additional advantages of being biomimetic and compliant, which reduces the chance of injury to the user. There are several types of artificial muscles available, such as shape memory alloys (SMAs), pneumatic artificial muscles (PAMs), and twisted coiled actuators (TCAs).

SMAs are thermal actuators that alternate between two states upon heating and cooling. They can be formed into artificial muscles with low voltage requirements and high power-to-mass ratios. Unfortunately, they have large amounts of hysteresis which makes them difficult to control, and their cycle life decreases as the amount they are strained increases (9).

PAMs are fabricated by covering an internal air bladder in a woven mesh such that when the air bladder is inflated, the system contracts (10). PAMs are advantageous as they are naturally compliant and they have a high power-to-weight ratio when the air compressor is ignored. Their main disadvantage is that they require an air compressor to actuate, which is loud, heavy, and frequently tethers the user to one spot. Recently, these muscles have been embedded into fabric to create a glove to help finger flexion (11,12).

Finally, TCAs are a promising actuator for wearable robotic devices due to their inherent compliance, low profile, easy and inexpensive fabrication method, and linear actuation (13). These artificial muscles are created by super-coiling silver-coated nylon thread, and they can carry loads up to 80 MPa. They will contract up to 21% when electrically heated and extend upon cooling. Unfortunately, their low bandwidth with passive cooling (0.03 Hz) limits their effectiveness in devices designed for rehabilitation purposes (14). To increase the potential for TCAs to be used in wearable robotic devices, their cooling time needs to be decreased.

Previous work has demonstrated that the cooling time is significantly impacted by the environment that surrounds the TCA. Common solutions have been to coat the TCA in hydrogel, surround it with water, or employ forced air convection (15,16). Of these solutions, a hydrogel coating increased the cooling rate the most, although the coating only lasted for 30 actuation cycles. Similar cooling rates have been achieved with forced convection of air and still water, however, when the TCA was surrounded by water it required approximately twice the input power to achieve a stroke of 10%.

Forced air convection has been implemented by running a computer fan near the TCAs or by placing a TCA in a rigid plastic tube and applying compressed air. However, neither of these solutions have been specifically designed for implementation in a soft wearable robotic device (16,17). Portable, wearable robotic systems have several design constraints unique to their applications. The systems must be lightweight and have minimal components, as the user must be able to carry the apparatus on their body. Additionally, the system should be flexible, compliant, and minimize any protrusions to ensure user comfort and decrease risk of injury.

Thus, this research presents a novel cooling apparatus for TCAs that is specifically designed for soft wearable robotic devices. It consists of a fabric channel to house the TCA and a miniature air pump to provide forced convection. Previous work performed simulations on this design, however, discrepancies between the simulation and experimental results indicated that additional experimental work would be valuable (18). In this paper, Section 2 outlines the design process and final design for the novel cooling apparatus. Section 3 describes the experimental apparatus, methodology, and results obtained for an experiment that investigates the impact of changing the dimensions of the channel on the performance of the TCA (Phase I). Section 4 documents a follow-up experiment that was completed to compare the performance of the TCA with and without the novel cooling apparatus (Phase II). Finally, Section 5 discusses study limitations, sources of error, and future work.

## 2. Channel design

The primary purpose of the cooling apparatus is to increase the cooling rate of the TCA, however, it should also facilitate the integration of TCAs into soft wearable robotic devices. To meet these objectives, several design requirements were considered during the development of the channel. First, the cooling apparatus had to be portable and help protect the user from the temperatures reached by the TCAs (up to 120${}^{\circ }$C). Next, the mass and size of the cooling apparatus needed to be minimized to ensure that the wearable device is as unobtrusive as possible. Ideally, the apparatus will also be durable, flexible, elastic, and breathable, to allow the user to comfortably wear it for extended periods of time. Finally, any negative effects of the cooling apparatus on TCA behavior (e.g., decreased stroke), should be minimized where possible to maintain the capabilities of the TCA.

### 2.1. Cooling mechanism

While considering the above objectives, the first decision made was how to decrease the cooling time of the TCA. This could have been accomplished by adding a coating to the surface of the TCA to increase its thermal conductivity or by changing the environment in which the TCA resides. Modifying the TCA by adding a coating was disregarded as an initial solution, since coatings increase the manufacturing complexity and cost of the TCA, they do not provide a means of integrating the TCA into a wearable system, nor would they help protect the user against the high temperatures reached by TCAs. Thus, the environment of the TCA was changed by enclosing a TCA in a channel and employing forced convection.

Forced convection could be accomplished with a liquid (e.g., water) or a gas (e.g., air). Water and other liquids have a significantly higher thermal conductivity compared to air, which would drastically reduce the cooling time of the TCA. However, liquid coolants have many disadvantages when considering them for a wearable device. They are denser than air and require additional hardware, such as a reservoir and valves, resulting in a significantly heavier and more complex system. Additionally, there is the risk of leaks, which could decrease user comfort, and nylon TCAs will absorb water over time, degrading their performance (14,19). Finally, the higher thermal conductivity of liquids results in higher power requirements for the heating phase of the TCA (15). Some of these disadvantages could be alleviated by surrounding the TCA in stagnant water, however this was disregarded as a potential solution as the temperature of the water would gradually increase with prolonged use of the TCA, and similar cooling times can be obtained with forced air convection (15). The main disadvantage of forced air convection is that air compressors are noisy, however, the advantages of less mass, hardware, and no degradation of TCA performance allow it to meet the requirements better than a liquid system.

There are three main options to circulate air: fans, air pumps, and air compressors. Air compressors were rapidly discarded as potential solutions, as their large size and mass limit their portability. Table 1 displays some commercially available miniature air pumps and fans. Air pumps are capable of producing higher air pressures than fans, however, fans have the advantages of being lighter, smaller, and producing more air flow than the pumps. From the options listed in Table 1, fans have the additional benefit of producing less noise (between 15–36 dB) when compared to the pumps (around 55 dB). For context, the volume of a normal conversation occurs at around 60 dB (20).

**TABLE 1:** Commercially available miniature air pumps and fans that meet the 20 mm height constraint.

Fan/pump	Company/part	Maximum air flow (cm${}^{3}$/min)	Maximum Pressure (atm)	Height (mm)	Length (mm)	Width (mm)	Mass (g)
Pump	Binaca SX-1	400	0.39	8	32	18	8
	Schwarzer precision SP 12 RO	650	0.49	12	51	27	23
	Schwarzer precision SP 16A RO-DV	1,500	0.79	16	56	36	38.5
	Binaca CX1	1,000	0.69	17	45	27	32
	Schwarzer precision SP 18A RO-D	2,500	0.59	18	62	32	44
Fan	Sunon fans UB393-700TC	1,170	0.00016	9	9	3	0.91
	Nidec copal electronics F16FB-05LLC/E	11,890	0.00005	16	16	4.5	1.2
	Delta electronics BSB0205HP-00EFG	16,140	0.00098	17	17	5.3	1.81
	Sunon fans MF20100V1-1000U-A99	53,800	0.00071	20	20	10	4.65

While fans would be the optimal solution due to their advantages, testing with preliminary prototypes using a Sunon Fans MF20100V1-1000U-A99 demonstrated that their low pressure capabilities were unable to force air through the devised solution. Therefore, a pump was selected by maximizing the air flow and pressure capabilities while minimizing the height and mass. From this trade off, the Schwarzer Precision SP 16A RO-DV pump was selected, and is the pump used in all of the experiments described in this paper.

### 2.2. Channel characteristics

The next step was to devise a method of enclosing the TCA that was flexible, easy to integrate into wearable devices, and could protect the user. It was decided to create a fabric channel, as fabric is flexible and can be sewn onto other materials, including insulation and underlying garments. The fabric for the channel must meet several criteria to ensure the success of the design. It should be flexible, durable, and be able to withstand temperatures above 120${}^{\circ }$C. Ideally, the material would be able to slightly stretch and be breathable, to ensure user comfort, and be able to hold a shape without additional supports, to minimize the complexity and cost of fabrication.

Two common durable fabrics are tightly woven nylon and polyester, which are often used in parachutes and windbreakers. While they both have a melting temperature above 220${}^{\circ }$C, nylon was selected because nylon threads have a lower coefficient of dynamic friction and a similar coefficient of static friction when compared to polyester (21). The dynamic coefficient of friction of nylon 6 is 7.6–22.2% lower than that of polyester, and the coefficient of static friction ranges between 3.6% smaller, up to 7.9% larger, depending on the circumstances. Tightly woven nylon 6 pack cloth with a thickness of 0.3 mm was sourced from Trident Textiles Corp. The material is 100% nylon with a 0.05 mm coating of ether-based thermoplastic polyurethane (ether TPU) on one side. The channels were created with the ether coating on the outside surface as it seemed to have higher friction, and there were concerns about melting the ether TPU. Ester TPU was selected as an alternative material as it has a melting temperature of approximately 145${}^{\circ }$C, it can stretch slightly, and it was used in another study to create PAMs embedded in wearable devices (12,22). Samples of 0.3 mm ester TPU were obtained from Plastic Film Corporation. Preliminary prototypes proved that both materials were easy to sew and were capable of maintaining their cross-sectional shape. To ensure that they could withstand the temperatures reached by TCAs, a TCA was held on the material at approximately 115${}^{\circ }$C for 10 min. There was no visible deformation to the nylon fabric, however, sections of the ester TPU melted. Thus, the material for the channels was selected to be nylon pack cloth.

**FIGURE 1:**
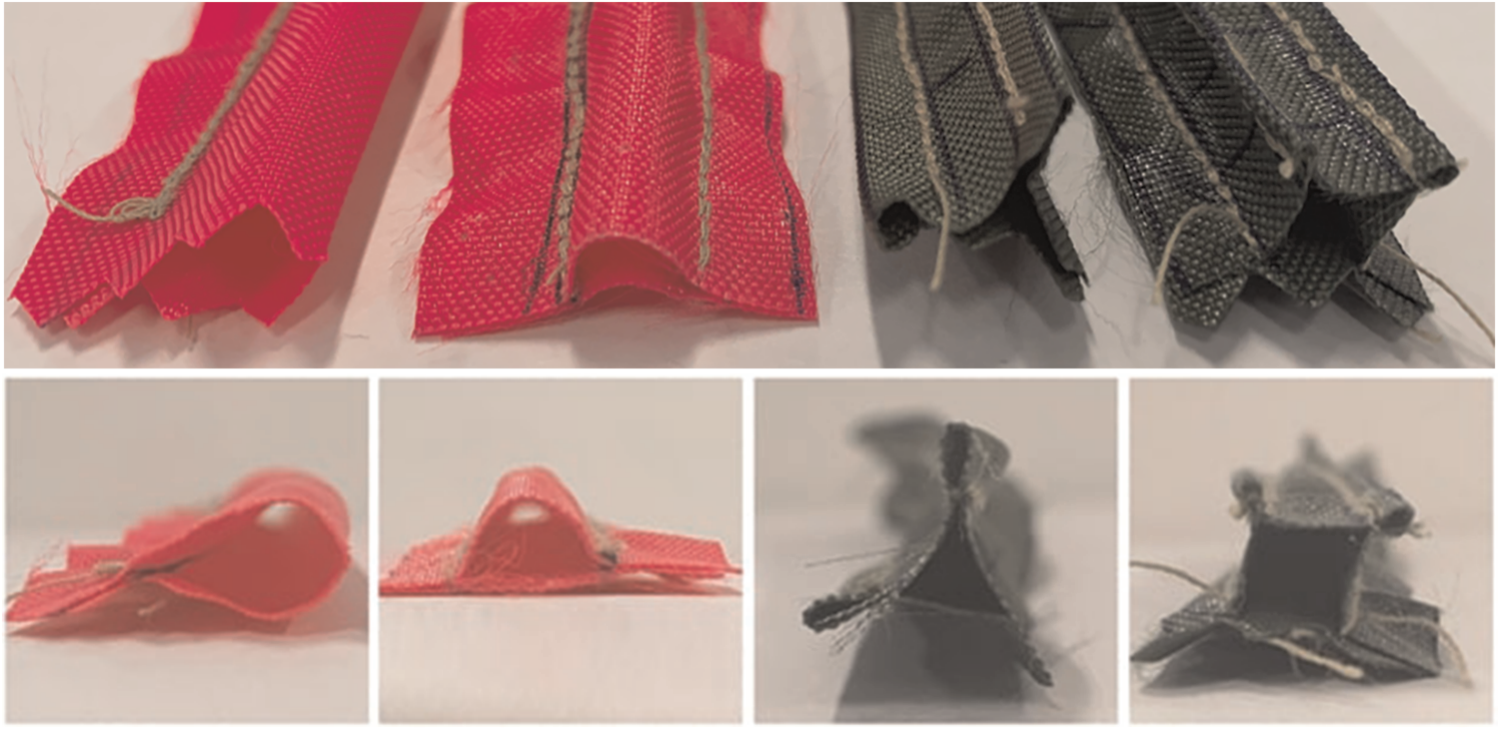
The top view (top) and front view (bottom) of the preliminary channel prototypes to test different channel shapes. From left to right the shapes tested include the following: circle, semi-circle, triangle, and square.

After the material was selected, preliminary prototypes were created to determine the cross sectional shape of the channel. The nylon cloth was sewn into four shapes—a circle, a semi-circle/ellipse, a triangle, and a square (Figure 1). The channel must be able to maintain its shape to ensure that there is adequate space for air to flow around the TCA and that no additional friction is caused by contact between the TCA and channel walls. It should also return to shape if deformed to ensure that the performance of the channel does not degrade if the user bumps their limb or folds the fabric. The circle was the easiest channel to manufacture; however, it was easy to flatten and did not return to its shape on its own, which resulted in the TCA being pinched by the fabric on two sides. The triangle was unable to remain open to provide an airway for the TCA. While the square and the semi-circle both had acceptable airways and could quickly return to their shapes when flattened, the semi-circle was significantly simpler to manufacture. For the rest of this paper, the channels being designed and tested will be semi-elliptical in shape. The main design parameters will be the width of the channel (the horizontal diameter of the ellipse) and the height of the channel (the vertical radius of the ellipse).

It is desirable to keep the dimensions of the channel as small as possible to reduce both the protrusion from the limb and the space on the device that would not be breathable, as the selected material is air impermeable. Additionally, if either the height or width are much larger than than the other, the ability of the channel to maintain its shape decreases. The minimum height and width were determined by considering the size of the TCA. The diameter of a 4-ply TCA is 1.6 mm, however the crimps used at the ends of the TCAs have a diameter of approximately 3 mm. Some additional space was required for the temperature sensor leads and air inlet. Thus, the smallest channel height and width were 4 and 6 mm, respectively.

Since the ideal height and width of the channel were unknown, an experiment (Phase 1) investigated the impact that changing these factors had on the performance of the TCA. Combinations of three heights and three widths were tested to determine if there was an effect from the height, the width, or the overall cross-sectional area (CSA) of the channel. Trial and error in making different sized channels revealed that there is some degree of variance in the size of the channel, even when the same fabrication procedure is followed. Thus, the size step was set to 2 mm to ensure that the sizes were distinct. The channels will be described as width-by-height, i.e., a 6×4 channel has a width of 6 mm and a height of 4 mm. The final channel sizes that were tested are the following: 6×4, 6×6, 6×8, 8×4, 8×6, 8×8, 10×4, 10×6, 10×8. The channel dimensions were kept as small as possible to minimize how much the channel would protrude, while still ensuring there were distinct differences between the channel sizes.

**FIGURE 2:**
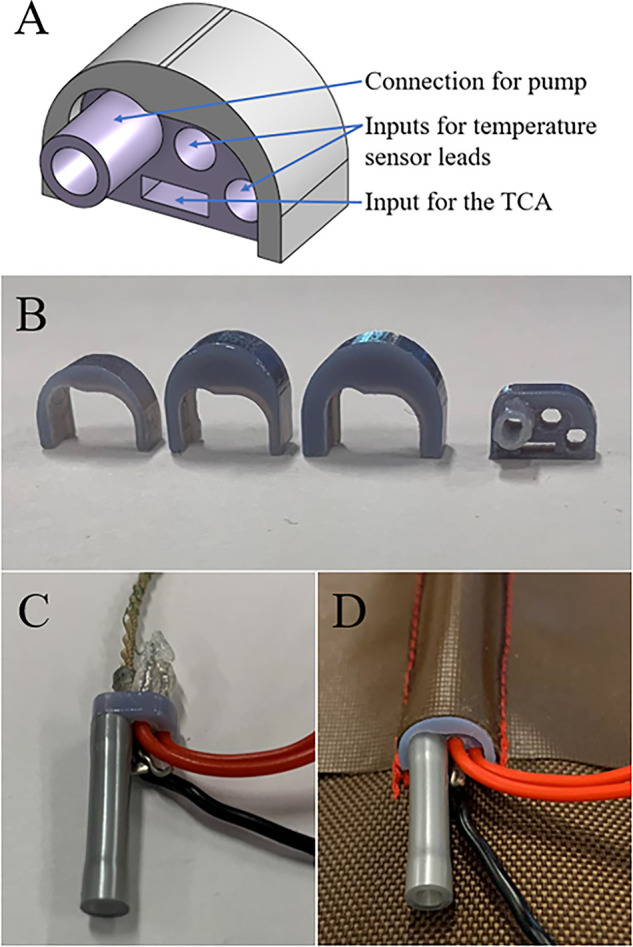
Inlet design (**A**) in CAD, (**B**) printed with different sized covers, (**C**) with the components connected, and (**D**) in a channel.

Prior to beginning the experiment, an inlet piece was designed to connect the channel, air pump, and TCA (Figure 2). It is composed of two parts: a small, central piece to attach all of the components, and a cover for the central piece that simplifies changing the size of the inlet to match the size of the channel. At this stage, the inlet was not permanently fixed to the channel to facilitate testing the channels with multiple TCAs. Instead, the inlet was deliberately created 2 mm wider and 0.5 mm taller than the desired dimensions of the channel and it was wedged into the channel inlet. The channel inlet was also created slightly larger than the body of the channel to accommodate the inlet.

**FIGURE 3:**
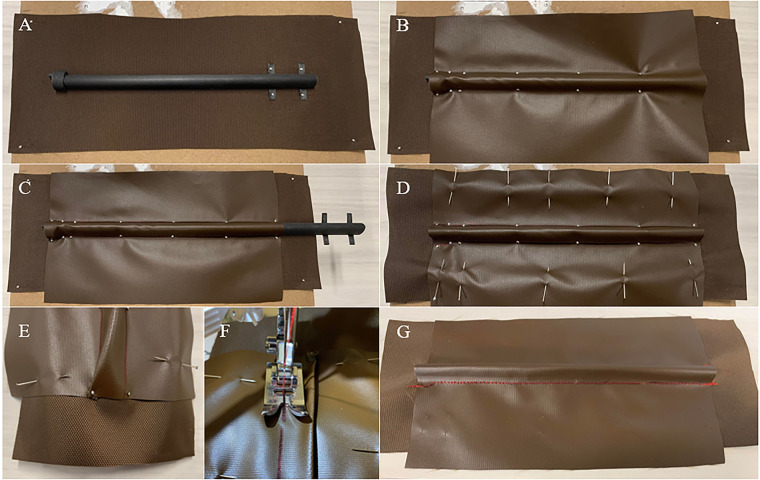
Channel fabrication procedure. First, the bottom and top layers are sized such that they are wider than the desired channel size to ensure that there is enough fabric to pin the material. The bottom layer is also sized longer than the desired length to simplify sewing. Then the following steps can be completed to create a channel: (**A**) The bottom layer and channel template are pinned in place to keep them from moving, and the inlet is placed at the end of the template. For simplicity, this is performed on a cardboard box to allow the pins to be pushed completely through the fabric. (**B**) The top layer of fabric is placed over the inlet and template, pulled taut, and pinned in place. (**C**) Before moving the template, the edge of the template is marked with pen as a guiding line for sewing. The template is slightly removed from the channel (to prevent it from getting trapped), and the end of the channel is pinned in place. (**D**) The two layers of fabric are pinned together to prevent them from slipping, and the channel is removed from the box. (**E**) To sew the channel, it must be flat along the seam. The fabric can be pulled taut and pinned in place at the outlet to help hold the opposite seam flat while starting to sew. (**F**) The seam is sewn along the guideline, while keeping the material pushed to the opposite side. (**G**) The previous two steps are repeated along the other side of the channel to obtain the finished product.

The channel fabrication process is outlined in Figure 3. To consistently create the channels, templates were designed and printed. Preliminary experimentation revealed that the channels were consistently wider and shorter than the desired dimensions, thus the templates were deliberately created 1 mm narrower and 0.5 mm taller. The channels were made to be 150 mm in length to accommodate TCAs that are 140 mm in length. The length of the channel was set to be approximately 10 mm longer than the loaded length of TCA to ensure that the entire TCA remained covered, as the TCA will creep slightly during use.

Finally, the channel on its own does not have enough insulation to protect the user from the temperatures reached by the TCA (up to 120${}^{\circ }$C). The pain threshold for hot temperatures has been shown to be between 42${}^{\circ }$C to 44.6${}^{\circ }$C (23,24), thus additional insulation will be required to keep the user comfortable. The primary parameter when selecting insulation is the thermal conductivity, as this will dictate how thick the insulation must be to protect the user. Other properties were considered for the purpose of a wearable device, such as the water absorption (in case the user sweats) and density. Initially the Alpha SmartTemp Liner for prostheses was used, as the material is specifically designed to be comfortable for users when in contact with their skin for extended periods of time. This solution was tested by holding a TCA onto the material at approximately 110${}^{\circ }$C for 5 min. It was found that two 8 mm layers were required to keep the temperature below 40${}^{\circ }$C. Thus, alternative options were investigated.

While several types of insulation were considered, mineral wool was selected due to its low thermal conductivity and density. Samples of 3 mm mineral wool with a foil backing were obtained from Insultech Inc. When the foil side of the insulation was placed facing the TCA, the 3 mm insulation was sufficient to protect the user from the temperature of the TCA. For integration in a wearable device, there would have to be an additional layer underneath the insulation, as the insulation will pull apart from itself if left uncovered.

**FIGURE 4:**
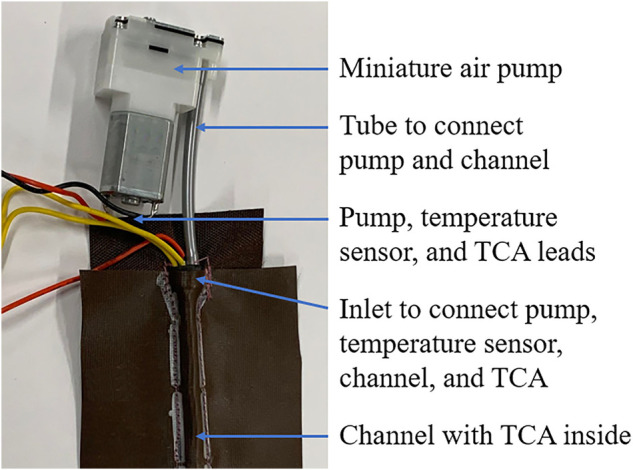
Final design of the cooling apparatus. The design consists of a fabric channel to house the TCA with a miniature air pump connected to the channel with a flexible plastic tube.

To summarize, the general design for the fabric channels is displayed in Figure 4. The channel can be fixed to a wearable device by sewing it to an underlying garment, and the TCA can move freely inside the channel. Thus, the channel can both provide a means of cooling the TCA and a means of assembling it into a system. One end of the TCA was fixed to the channel inlet using the inlet piece described above, and the free end was coupled to its load using a thread as an artificial tendon. A flexible power line was also fixed to the free end of the TCA to allow the TCA to be actuated using Joule heating.

## 3. Phase I: impact of channel height and width on TCA performance

### 3.1. Phase I: methods

After the channel design was finalized, the impact of channel height and width on the performance of the TCA was investigated. This was accomplished by actuating TCAs in the nine channel sizes described above: 6×4, 6×6, 6×8, 8×4, 8×6, 8×8, 10×4, 10×6, and 10×8. TCA performance was assessed by comparing the cooling time, heating time, and stroke of the TCA in each channel, as these metrics are often used when evaluating the performance of thermal actuators (15,16,25,26). For this experiment, the cooling time was defined as the time it takes the TCA to cool from 100${}^{\circ }$C to 35${}^{\circ }$C; the heating time was defined as the time to heat the TCA from 23${}^{\circ }$C to 100${}^{\circ }$C; and the stroke was defined as the difference between the maximum displacement of the TCA and its position when it returned to room temperature after the heating–cooling cycle. The cooling time was stopped at 35${}^{\circ }$C because preliminary experimentation found that the air output from the pump increases in temperature and can reach up to 30.2${}^{\circ }$C.

**FIGURE 5:**
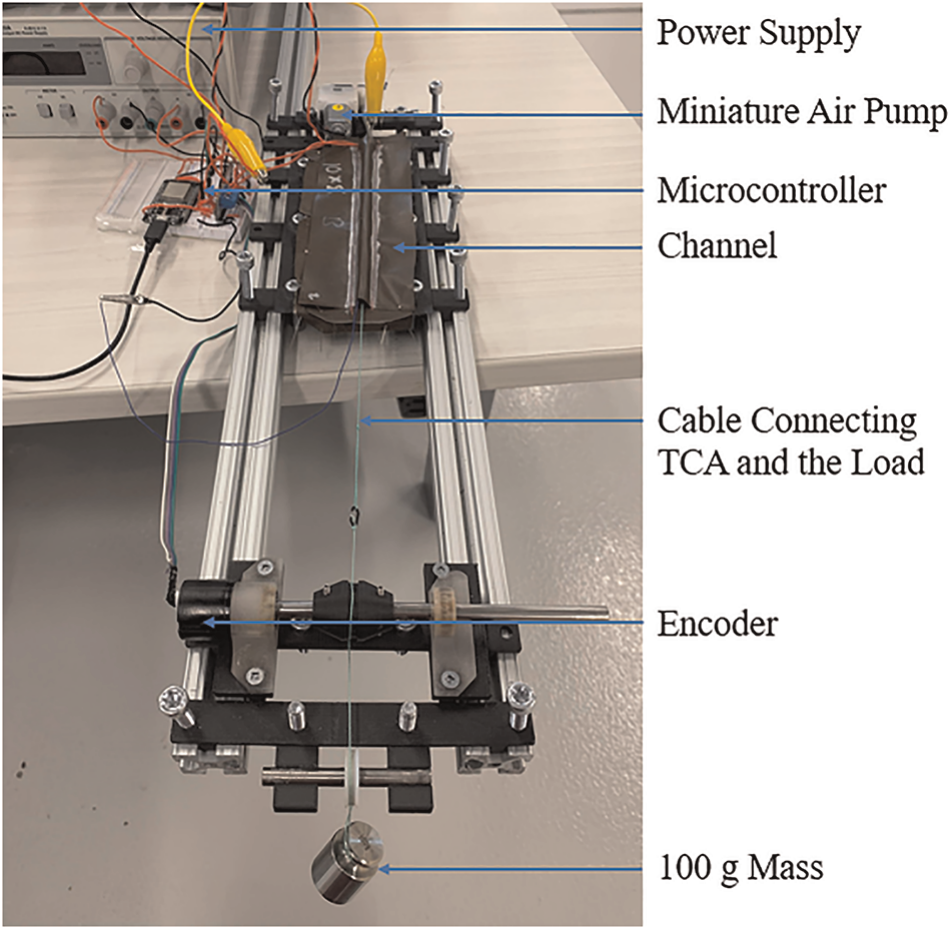
The experimental apparatus used to collect data.

The experimental apparatus used for data collection is displayed in Figure 5. Insulation was placed on the platform on which the TCA and the channel rested to mimic the construction of a wearable device. The TCA was attached to a previously developed module that combines pulse width modulation circuitry, a current sensor (ACS70331EESATR-005U3), and a temperature sensor into one circuit board (14). A resistive temperature sensor detector consisting of a 40 AWG shielded copper wire wrapped around the TCA was used, since the temperature sensor must be able to fit within the channel without easily falling off, or compromising the ability of the TCA to move within the channel (14). The temperature sensor was located at the fixed end of the TCA to reduce the motion that could dislodge the sensor. The displacement of the TCA was measured using an encoder (AEAT-6012-A06) by fixing a string to the free end of the TCA and wrapping it around a pulley that was attached to the encoder. Set screws were used to prevent slipping between the string, the pulley, and the shaft. A 100 g mass was hung from the other end of the string to keep the TCA in tension.

The TCAs were made using a previously developed methodology (14). Four-ply silver-coated nylon thread was obtained from VTechnicalTextiles (Part number: 260151023534) and was supercoiled with a load of 165 g. The coiled thread was plyed by folding it in half to prevent untwisting and the ends of the TCA were crimped to prevent the TCA from fraying using standard terminal crimps obtained from McMaster-Carr (Part ID: 69525K47). The untrained TCA length was 85 mm, and it was trained by stretching the artificial muscle 4–5 mm, holding it in position and heating the TCA at approximately 3 W until it reached 140${}^{\circ }$C. This process was repeated until the TCA was 110 mm (around 30% longer), which resulted in a final, unloaded TCA length of 138 mm, as the crimps add 14 mm on either side of the TCA.

To collect the data, the TCA was centered in a channel, then heated from 23${}^{\circ }$C to 100${}^{\circ }$C at an average power of 2 W. The power was regulated to ensure that the heating time could be compared between samples, which was accomplished by controlling the duty cycle of the input voltage (12 V) to account for the change in resistance of the TCA. The resistance of the TCA was computed using R=V/I, where R is the electrical resistance of the TCA, V is the average voltage applied to the TCA, and I is the average current through the TCA. Once the temperature of the TCA reached 100${}^{\circ }$C, the power input to the TCA was stopped, and the miniature air pump was turned on at 12 V. The pump was left on until the TCA reached 35${}^{\circ }$C, at which point the TCA was left to cool to room temperature passively, and the displacement of the TCA at room temperature was recorded before starting the next trial. This process was repeated three times before changing the channel. In total, 81 data points were collected at each channel size. To account for variability in the fabrication process, these repetitions were split evenly among three channels of the same size and three TCAs. The data collection process was blocked by TCA, i.e., all of the data were collected with TCA 1 before using TCA 2 or TCA 3.

**FIGURE 6:**
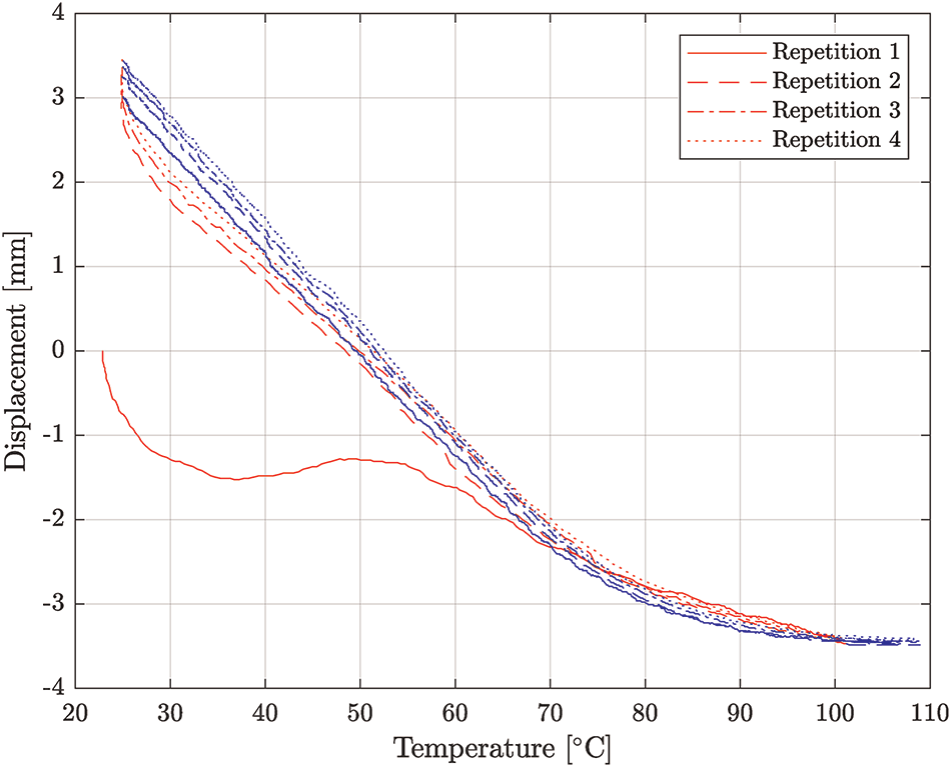
Four consecutive cycles of TCA heating from 25${}^{\circ }$C to 100${}^{\circ }$C after not being used overnight. The red and blue represent the heating and cooling portions of the cycle, respectively. The heating curve of the first repetition is significantly different from the rest of the trajectories.

Additionally, a warm-up procedure was completed if the TCA had not been used for more than 10 min, as it was noticed that the heating temperature–displacement curve for the heating cycle was significantly different if the TCA had not been used overnight, as seen in Figure 6. The warm-up procedure consisted of heating and cooling the TCA between 23${}^{\circ }$C and 100${}^{\circ }$C until the difference between the starting and ending positions at 23${}^{\circ }$C was less than 0.25 mm. This took 2–4 cycles, depending on how long it had been since the TCA was last used.

### 3.2. Phase I: results

After the data were collected, a statistical analysis was performed for each of the three parameters (cooling time, heating time, and stroke) to determine if there were differences between the channel sizes. First, the data were assessed to determine if there were outliers by calculating the studentized residuals and determining if any of the residuals were greater than ±3. Although the cooling time data had five outliers, it was not possible to remove them from the analysis, as there was no apparent reason for the variation. The heating time data had one outlier, which was removed, because in the immediate trial afterwards, the power line broke, and therefore it is very likely that the power line was already partially broken. The stroke data had no outliers. Then, the normality of the data was assessed using the standardized residuals with the Kolmogorov–Smirnov test, as the sample size was larger than 50. None of the data were normally distributed, thus Friedman tests were performed to determine if differences existed between the channels. The results of the Friedman tests stated that channel size had a significant effect on all three parameters, with p<0.001. *Post hoc* testing was performed using the Wilcoxon test, and a Bonferroni correction factor of 36 was applied to the pvalues to account for accumulated error from multiple comparisons, as 36 comparisons were performed for each parameter. The correction factor was applied by multiplying the pvalue by 36, to allow the significance threshold to remain at 0.05. The p values reported are those obtained after the correction factor was applied and the p values for all of the comparisons are reported in Table S1.

First, the data were analyzed to determine which channel resulted in the best TCA performance. Ideally, one channel would have the lowest cooling and heating times and the highest stroke, however, the best channel is defined as the one that balances these three parameters. The best channel cannot simply be the one with the lowest cooling time if it also greatly increases the heating time or decreases the stroke, as this would have negative implications for a wearable device. Significant increases in heating time indicate that the channel causes the TCA to be less efficient, which would negatively impact the battery lifetime of a wearable device. Likewise, decreases in TCA stroke would negatively impact the performance of the device, as there would either be a lower achievable range of motion, or a longer TCA would be required to obtain the original stroke.

**FIGURE 7:**
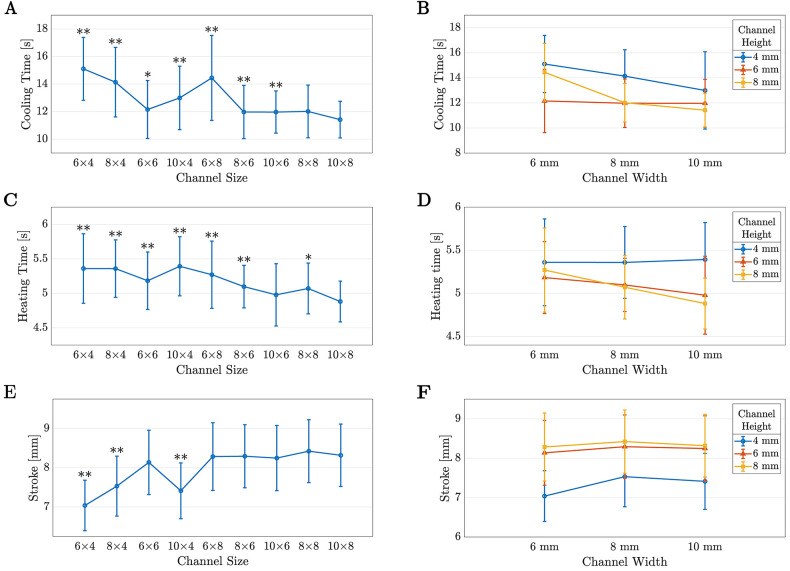
The means and standard deviations for the data from Phase I plotted with respect to increasing cross-sectional area (**A**, **C**, **E**) and height and width (**B**, **D**, **F**). In (**A**), (**C**), and (**E**), statistically significant differences with the 10×8 pouch are marked by an ∗ for p<0.05 or ∗∗ for p<0.001.

The descriptive statistics for each parameter are summarized in Table 2. To illustrate them, Figure 7A,C,E plot the data with respect to increasing cross-sectional area (CSA), with the statistically significant differences for the 10×8 channel marked. It can be seen that for the cooling time, 10×8 is statistically different from all of the channels except 8×8 (11.42±1.33 s vs. 12.02±1.91 s, p=0.051), and for the heating time, 10×8 is significantly different from all of the channels except for 10×6 (4.88±0.3 s vs. 4.98±0.45 s, p=1). Furthermore, there is no statistically significant difference between the stroke for 10×8 and any channel with a height greater than 4 mm. Thus, it can be concluded that for a 4-ply TCA, the best channel out of the sizes present in this experiment is 10×8, as that channel has a good balance between cooling time and heating time, and a comparable stroke to the other sizes. While the cooling time for 10×8 is not statistically different from 8×8, 8×8 has a statistically higher heating time. Similarly, the heating time for 10×8 is not different from 10×6, however the cooling time for 10×6 is higher.

**TABLE 2:** Descriptive statistics for the parameters that were assessed to compare TCA performance in channels with different dimensions.

Channel		6×4	8×4	6×6	10×4	6×8	8×6	10×6	8×8	10×8
Cooling time (s)	Mean	15.11	14.14	12.16	13.00	14.45	11.98	11.97	12.02	11.42
	Standard deviation	2.28	2.52	2.10	2.30	3.08	1.93	1.53	1.91	1.33
	Median	15.30	13.70	12.35	12.64	14.46	12.16	12.25	11.87	11.30
Heating time (s)	Mean	5.36	5.36	5.18	5.39	5.27	5.10	4.98	5.07	4.88
	Standard deviation	0.50	0.42	0.42	0.43	0.49	0.31	0.45	0.37	0.30
	Median	5.21	5.33	5.18	5.27	5.23	5.09	4.91	5.02	4.89
Stroke (mm)	Mean	7.04	7.53	8.14	7.41	8.29	8.29	8.25	8.42	8.32
	Standard deviation	0.64	0.76	0.82	0.71	0.86	0.80	0.83	0.80	0.80
	Median	7.11	7.59	8.07	7.43	8.31	8.18	8.37	8.55	8.34

Channels are ordered from left to right by increasing cross-sectional area.

Next, the effect of width and height on the TCA performance was examined. To help visualize if there were any trends in the data based on height or width, the data were plotted with respect to these parameters in Figure 7B,D,F.

#### 3.2.1. Cooling time

Figure 7B illustrates that the impact of changing the width and height of the channel was different at each level of height and width. These simple main effects were tested using additional Friedman tests, which stated that height had a significant effect at each width (p<0.001 for all three) and width had a significant effect for heights of 4 and 8 mm (p<0.001). The effect of width at a height of 6 mm was not significant (p=0.391).

At all widths, channels with a height of 4 mm (the blue points) had the highest cooling times. When the height was increased to 6 mm (the red points), there was a statistically significant decrease in the cooling time (p<0.001 for all widths). An additional increase in height to 8 mm (the yellow points) resulted in the average cooling time either decreasing further (10 mm width, p<0.001), remaining approximately the same (8 mm width, p=1), or increasing (6 mm width, p<0.001). There was no significant difference between 6×4 and 6×8 (p=0.648).

When analyzing the effect of width on the cooling time, there is a decrease in average cooling time as the width increases at 4 and 8 mm heights. At 4 mm (the blue line), the decrease in cooling time is significant when the width increases from 8 to 10 mm (p=0.004), however it is not significant between 6 and 8 mm widths (p=0.468). The opposite effect is observed at the 8 mm height (the yellow line)—the increase in width is significant between 6 and 8 mm (p<0.001), yet it is not significant between 8 and 10 mm (p=0.051).

If the data are regarded as average cooling time as a function of CSA, there is a slight downward trend as the CSA increases, with a Pearson correlation coefficient of −0.739 and a slope of −0.071 s/mm${}^{2}$. Interestingly, the 6×8 and 8×6 channels have the same CSA, hence the stark difference between their mean cooling times (14.45±3.08 s vs. 11.98±1.93 s, p<0.001) highlights that the dimensions of the channel are important, not just the overall CSA.

#### 3.2.2. Heating time

Similarly, the heating time of the TCA slightly decreases as the channel CSA increases with a slope of −0.012 s/mm${}^{2}$. For this parameter, the Pearson correlation coefficient is −0.884. Again, the difference between the heating times for 6×8 and 8×6 (5.27±0.49 s vs. 5.10±0.31 s, p=0.008) show that the height and width have an impact on the heating time, and these trends are illustrated in Figure 7D.

From the simple main effect analysis for heating time, increasing the height of the channel did not have an effect when the width was 6 mm (p=0.177), yet there was an effect at widths of 8 and 10 mm (p<0.001 for both). *Post hoc* testing revealed that this effect was only present when the height increased from 4 mm (the blue points) to 6 mm (the red points), with p<0.001. There was no significant difference in the heating time when the height was further increased to 8 mm (the yellow point).

Likewise, the effect of width was not significant at a height of 4 mm (p=0.916), however, increasing the width produced a significant decrease in average heating time at heights of 6 mm (p=0.006) and 8 mm (p<0.001). At a height of 6 mm (the red line), the effect of increasing the width was only significant between 6 and 10 mm (p=0.003). However, at a height of 8 mm (the yellow line), the effect of width was significant at both step increases, with p=0.006 for 6 to 8 mm and p=0.002 for 8 to 10 mm.

#### 3.2.3. Stroke

The final parameter that was analyzed was the stroke of the TCA. Again, simple main effects were assessed using the Friedman test and the trends are shown in Figure 7F. The effect of height was significant at all three widths (p<0.001 for all three), and the effect of width was significant at a height of 4 mm (p<0.001). *Post hoc* analysis revealed that the effect of height was only significant with the increase from 4 mm (blue points) to 6 mm (red points), with p<0.001 at all three widths. There was no significant effect on the stroke when the height was further increased to 8 mm. Similarly, the effect of width at 4 mm of height (the blue line) was only significant when the width increased from 6 to 8 mm (p<0.001). Thus, it appears that the channel dimensions did not significantly affect the stroke of the TCA, provided that the channel height was greater than 4 mm.

### 3.3. Phase I: discussion

This experiment demonstrated that TCA performance varied as the height and width of the channel changed. As illustrated in Figure 7A, there was a slight decrease in mean cooling time as the CSA of the channel increased, however this decrease is influenced by the dimensions of the channel. To decrease the cooling time of the TCA, the thermal resistance between the surface of the TCA and the environment needs to be reduced. The equation of thermal resistance for convective cooling, $R_{conv}$, is displayed in Equation 1, where h is the convective heat transfer coefficient and $A_{TCA}$ is the surface area of the TCA that is exposed to forced convection (27). For a given TCA, the surface area would be fixed, thus to decrease the thermal resistance, h must be increased.


(1) $$R_{conv}=\frac{1}{hA_{TCA}}$$

The convective heat transfer coefficient depends heavily on the geometry of the hardware. For concentric cylinders, there is a complicated relationship between h and the diameters of the cylinders (27). It is hypothesized that the relationship between h and the dimensions of the channel would be even more complicated due to the half-elliptical shape and off-center placement of the TCA. However, for concentric cylinders, h is proportional to the input velocity of the fluid. It is assumed a similar relationship occurs with the channels—the cooling time will decrease as the input air velocity increases.

Fluid flow is also heavily dependent on geometry, and for laminar flow in a circular pipe, the flow resistance, $R_{\;flow}$, can be predicted using Pousuille’s Law, which is shown in Equation 2. Here η is the viscosity of the fluid, l is the length of the tube, and r is the radius of the tube (28). While this equation cannot be directly applied to the channel, the understanding that flow resistance decreases as the pipe radius increases is assumed to be applicable.


(2) $$R_{\;flow}=\frac{8\eta l}{\pi r^{4}}$$

The flow resistance will directly impact the fluid flow in a pipe, as for a fully developed laminar flow in a horizontal pipe, the flow, Q, is inversely proportional to the flow resistance and directly proportional to the pressure gradient across the pipe, ΔP, as seen in Equation 3 (28). It is also known that for pumps, there is an inverse relationship between the required pressure and the flow rate that the pump can output. Thus, given the limited pressure capabilities of the miniature pump, it is assumed that the cooling time decreased as the channel dimensions increased, because the lower flow resistance allowed the pump to provide a higher flow rate. This relationship was not linear, as the flow resistance, air velocity, and thermal resistance will depend on the specific geometry of the channel. Future work should further investigate and quantify this relationship to help optimize channel dimensions for variety of applications.


(3) $$Q=\frac{\Delta P}{R_{\;flow}}$$

**FIGURE 8:**
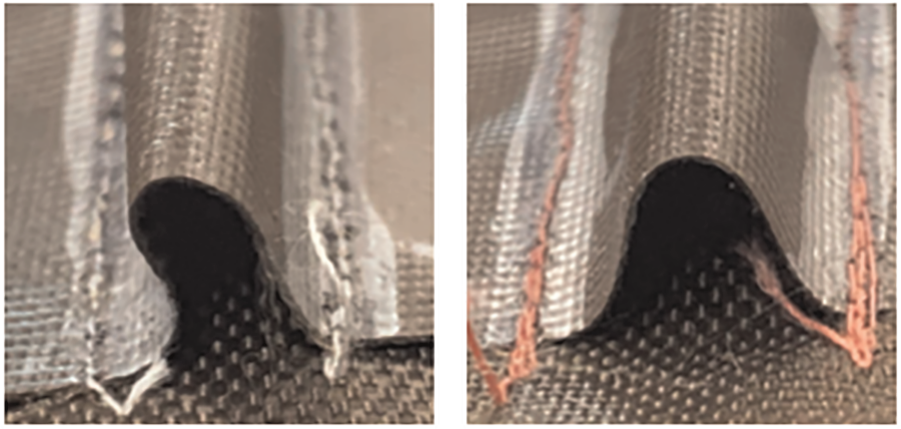
The approximate cross-sectional area shape for 6×8 (left) and 10×8 (right) channels. The sides of the 6×8 channel cave in compared to a channel where the width is larger than the height.

The effect that the specific height and width of the channel had on the cooling time of the TCA is highlighted by the contrast between the 6×8 and 8×6 channels. Despite the CSA being approximately the same, the cooling time for 6×8 was significantly higher and was the same as 6×4. This could be partially explained by observing the channel itself. The 6×8 channel was the only size where the height was larger than the width, and it was noticed that the sides of the channel had a tenancy to cave in slightly, as shown in Figure 8. This would reduce the effective CSA for the channel, increase the air resistance, and could increase the chance of the TCA coming in contact with the channel walls. Thus, it is recommended that the channel height should be the same or less than the channel width.

The geometry of the channel also influenced the heating time of the TCA. Unlike the cooling phase, a higher thermal resistance is better for the heating phase as this would reduce heat loss to the environment and reduce the heating time. As shown in Figure 7C, the heating time decreased as the CSA increased, for which a possible explanation is that the additional stagnant air in the larger channels acted as better insulation and allowed less heat loss to the environment. This is consistent with the results found when TCAs were heated in rigid plastic tubes; as the tube diameter increased, the heating time constant decreased (17).

A noticeable exception to this trend are the channels with a 4 mm height—there was no difference in the heating times between the 6×4, 8×4, and 10×4 channels. A possible explanation is that due to the low height, the TCA was in contact with the top of the channel, and thus there was heat loss through the channel to the surrounding environment. These channels also had the lowest strokes, which supports the prediction that the TCA was in contact with the channel at the 4 mm height as the stroke could be reduced due to additional friction from increased contact with the channel walls. The 6×4 channel had a lower stroke than 8×4 and 10×4, which is likely due to additional contact with the channel sides.

As illustrated in Figure 7F (red and yellow lines), the stroke was the same between the channels with 6 and 8 mm heights. This can be explained as once the TCA is no longer in contact with the top of the channel, there would be no additional frictional forces. The only significant difference occurred between 6×6 and 8×8 (8.14±0.64 mm vs. 8.42±0.80 mm, p<0.001). It is unclear why this occurred, as there was no difference between the heating time or cooling time for those two channels.

## 4. Phase II: effect of fabric channel on TCA performance

### 4.1. Phase II: methods

As concluded in Phase I, the channel height and width impact the performance of the TCA, and for a 4-ply TCA, the best size out of those tested was the 10×8 channel. An additional evaluation was completed to compare the performance of the TCA with and without the fabric channel to determine the efficacy of the channel in cooling the TCA and its impact on TCA performance. To accomplish this, TCA performance will be compared across the following four cases: (1) active cooling with the channel, (2) active cooling without the channel (current state-of-the-art), (3) passive cooling with the channel, and (4) passive cooling without the channel (basic operation).

This experiment was completed using the same apparatus as Phase I, which is described in Section 3.1. The experimental procedure was also very similar to that of Phase I, however two changes were made to be more consistent with how the TCAs and channels would be used in practice. The first change was to reduce the maximum temperature from 100${}^{\circ }$C to 85${}^{\circ }$C, as the temperature–displacement curve consistently flattened at around 85${}^{\circ }$C. This is likely due to the coils of the TCA coming in contact with one another and preventing further contraction. The second change was to immediately begin the next heating–cooling cycle once the TCA reached 35${}^{\circ }$C. Thus, the experimental procedure is summarized by the following steps: (1) the TCA was heated to 85${}^{\circ }$C, without any active cooling; (2) the TCA was cooled to 35${}^{\circ }$C, with or without active cooling (depending on the case); (3) Steps (1) and (2) were repeated five consecutive times; and (4) the data from the first repetition were discarded as the TCA was heated from room temperature, as opposed to 35${}^{\circ }$C. For each case, 96 repetitions were collected, which were split evenly among four TCAs and, where applicable, among three 10×8 channels (the same ones that were utilized in Phase I), to account for variability in the fabrication procedure. The order of the cases was randomized for each TCA, and the data were blocked such that all of the data were collected on one TCA before moving to the next.

To assess TCA performance, the cooling time, heating time, stroke, and maximum hysteresis were recorded and compared between the cases. For this experiment, the cooling time was defined as the time it takes to cool the TCA from 85${}^{\circ }$C to 35${}^{\circ }$C; the heating time was defined as the time to heat the TCA from 35${}^{\circ }$C to 85${}^{\circ }$C; the stroke was defined as the difference between the maximum displacement and the position of the TCA at 35${}^{\circ }$C at the end of the cooling phase; and the maximum hysteresis was defined as the largest difference in TCA position between the heating and cooling curves. The amount of hysteresis was computed by calculating the absolute difference between the position of the TCA at each point on the heating curve and the equivalent point (closest in temperature) on the cooling curve. The largest difference was recorded and was divided by the stroke for that trial, to express the maximum hysteresis as a percent of the stroke. Comparing the hysteresis as a percent of stroke reduces the possibility that a significant difference in the amount of hysteresis between cases exists solely due to a difference in TCA stroke.

### 4.2. Phase II: results

The descriptive statistics for the four parameters are displayed in Table 3. After the data were collected, they were checked for outliers using the studentized residuals. There were no outliers in the cooling time, heating time, or stroke data. There were two outliers present in the hysteresis data, however they were left in the analysis as there was no apparent reason for the variation. Then, the normality of the data were assessed using the Kolmogorov–Smirnov test on the standardized residuals. Once again, the data were not normally distributed, therefore Friedman tests were conducted to determine if a statistically significant difference existed in the data. The Friedman test revealed that there was a significant difference present for each parameter, with p<0.001 for cooling time, heating time, and stroke and p=0.041 for hysteresis. *Post hoc* testing was completed using the Wilcoxon test and a Bonferroni correction factor of 6 was applied to the p values in the same manner as Phase I to account for multiple comparisons. The adjusted p values for all comparisons are presented in Table S2.

**TABLE 3:** Descriptive data for Phase II.

Case		1. Channel active cooling	2. No channel active cooling	3. Channel passive cooling	4. No channel passive cooling
Cooling time (s)	Mean	12.54	8.57	34.92	21.71
	Standard deviation	2.31	0.71	1.48	1.24
	Median	12.17	8.68	34.90	21.72
Heating time (s)	Mean	3.76	3.61	3.52	3.46
	Standard deviation	0.71	0.69	0.66	0.71
	Median	3.50	3.41	3.39	3.32
Stroke (mm)	Mean	3.89	4.66	5.21	5.40
	Standard deviation	0.77	0.37	0.35	0.44
	Median	4.08	4.66	5.21	5.41
Maximum hysteresis (%)	Mean	14.06	12.59	11.23	11.60
	Standard deviation	6.30	3.19	2.38	2.15
	Median	12.02	12.20	11.42	11.56

**FIGURE 9:**
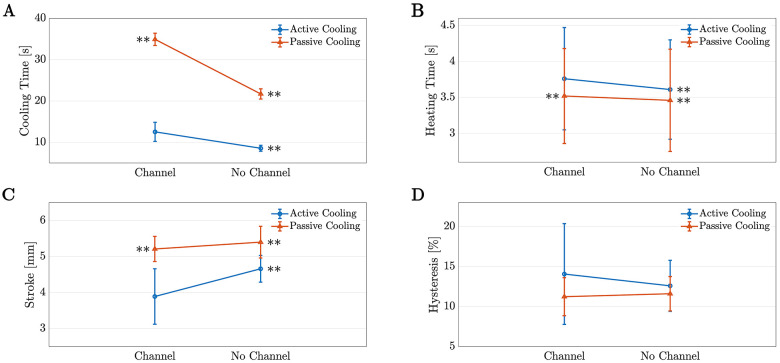
The means and standard deviations for the data from Phase II plotted for (**A**) cooling time, (**B**) heating time, (**C**) stroke, (**D**) and maximum hysteresis as a percent of stroke. The number adjacent to the point marks which case it is and if there is a statistically significant difference with Case 1 (channel, Active Cooling), it is marked by ∗∗ for p<0.001.

Figure 9 plots the data obtained in this experiment with the statistically significant differences for Case 1 marked. Interestingly, Case 1 is significantly different from all other cases for cooling time, heating time, and stroke, with p<0.001 for all comparisons. Conversely, there is no significant difference between the amount of hysteresis present in Case 1 and the other cases (p=1, p=0.120, and p=0.180, when compared with Case 2, 3, and 4, respectively), however Case 1 has approximately twice the standard deviation.

These data illustrate that the channel successfully reduced the cooling time of the TCA, however this occurred at a cost of increasing the heating time and decreasing the stroke. When the cooling time is compared to basic operation (Case 4, no channel with passive cooling), the cooling apparatus reduced the average cooling time by 42% (from 21.71±1.24 s to 12.54±2.31 s, p<0.001). The cooling time was also compared with the performance in a channel without active cooling (Case 3) to simulate the cooling time in a wearable device if active cooling was not employed. In this instance, the addition of active cooling reduced the cooling time by approximately 64% (34.92±1.48 s to 12.54±2.31 s, p<0.001). Finally, to complete the study, the cooling time of the TCA in the channel with active cooling (Case 1) was compared to the current state of the art: active cooling without a channel (Case 2). Unfortunately, the cooling time for Case 1 was larger than the cooling time for Case 2 (12.54±2.31 s vs. 8.57±0.71 s, p<0.001), however Case 2 is not feasible for wearable devices as the exposed TCA could burn the user or become caught on objects in the environment. A cooling time closer to that of Case 2 may be obtainable with further improvements to the channel design.

Ideally, improvements to the channel will also allow the TCA heating time and stroke to be closer to the values obtained without a channel. Surprisingly, the heating time of the TCA increased with the addition of the channel, however this difference was relatively small—approximately 4% between Cases 1 and 2, or 9% between Cases 1 and 4. A larger drawback is the decrease in stroke, as the addition of the channel caused reduced the stroke by approximately 19% between Case 2 and Case 1 (4.66±0.37 mm vs. 3.76±0.77 mm, p<0.001). Interestingly, the cases with passive cooling obtained higher strokes, even when the channel was present. With passive cooling, the addition of the channel reduced the stroke by 3.5% (5.40±0.44 mm vs. 5.21±0.35 mm, p=0.004).

### 4.3. Phase II: discussion

Unsurprisingly, the two cases with passive cooling had longer cooling times, and Case 3 (with the channel) had the largest cooling time. In Case 3, there would be minimal air circulation, and the air itself would also have to cool, since it would be warm from the end of the heating phase. Conversely, the other cases had some means of air circulation, whether it be from the pump when active cooling was incorporated in Cases 1 and 2, or natural circulation from the hot TCA being exposed to the environment in Cases 2 and 4. It is hypothesized that the cooling time for Case 2 was lower than that of Case 1 due to the lack of channel allowing constant natural convection (especially during the heating phase) in addition to the forced convection from the pump. Natural convection would provide an advantage, as the air that is heated during the heating phase can immediately move away from the TCA, as opposed to being trapped by the channel, reducing the surrounding temperature. Additionally, Case 2 likely has a higher air flow rate than Case 1, as there is no added flow resistance from the channel.

It is also possible that there was additional air flow in Case 2 due to air entrainment. Air entrainment is the phenomenon of air in the environment being pulled (entrained) along the air stream due to the pressure gradient, which increases the total air flow. Air entrainment would be prevented when the channel was used, as the inlet was sized such that the channel was sealed. Thus, future work could investigate improving the channel design by not sealing the inlet of the channel, to allow air entrainment, or by adding small holes in the channel, to allow natural convection.

One of these modifications may be sufficient to reduce the heating time with a channel to what it was without a channel, as the difference was less than 10%. The increase in heating time with the addition of the channel was unexpected, as it was predicted that the stagnant air inside the channel would act as insulation during the heating phase and allow the heating time to be reduced, as natural convection would not occur to the same extent. However, it is possible that the difference in heating times occurs from the addition of active cooling, not the channel. The difference in mean heating time with and without active cooling is 0.24 s with the channel and 0.15 s without the channel. In contrast, the difference in mean heating times with and without the channel is 0.15 s for active cooling and 0.06 s for passive cooling.

While the difference in heating time with the channel present was small, the addition of the channel and active cooling greatly reduced the stroke of the TCA. This likely stems from a combination of additional friction (when compared to Case 2) and a shorter cooling time (when compared to Case 3). The shorter cooling time would negatively affect the stroke, as preliminary experimentation found that the TCA, when loaded, has a tendency to slowly creep. Thus, the cases with passive cooling achieved higher strokes as the TCA has more time to extend, and it is predicted that this is the reason why the stroke for Case 3 is comparable to that of Case 4, despite the addition of the channel. On the other hand, the stroke for Case 2 is smaller than those of Cases 3 and 4, as the TCA does not have time to creep.

**FIGURE 10:**
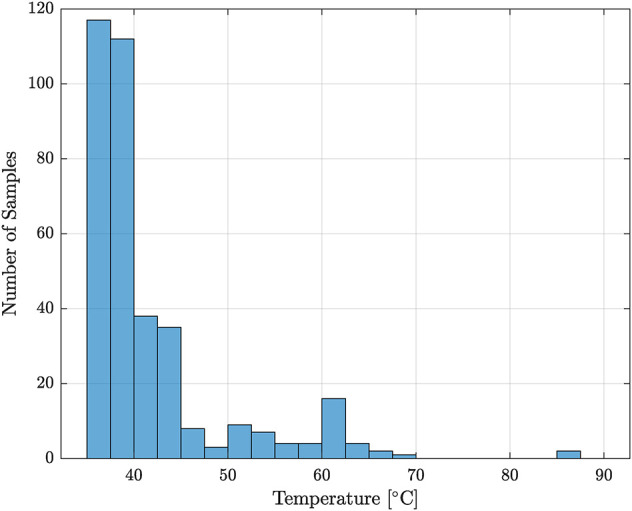
The distribution of temperatures at which the maximum hysteresis occurred.

Despite the reduction in stroke, there was no significant difference in the amount of hysteresis present between Cases 1 and 2. There was also no significant difference in the hysteresis between Cases 3 and 4 (p=0.648), indicating that the addition of the channel does not increase the amount of hysteresis present in the system. However, the standard deviation for Case 1 has approximately twice the standard deviation compared to the other cases, suggesting that the behavior of the TCA may be harder to predict and model, as there is more variation. Interestingly, the majority of the samples had the maximum hysteresis occur between 35–40${}^{\circ }$C, as seen in Figure 10. A possible explanation is that when the power is turned on, the sudden increase in temperature causes a quick contraction, however when cooling, the progression over the same positions occurs more gradually. This is likely related to the heat transfer rates, as at the end of the cooling cycle, the reduced temperature differential results in reduced heat transfer.

Finally, a brief comparison between the results for the 10×8 channel with active cooling from Phase I and Phase II was performed. As expected, the average heating time and stroke were higher in Phase I than Phase II (4.88±0.30 s vs. 3.76±0.71 s, and 8.32±0.80 mm vs. 3.89±0.77 mm, respectively), as the temperature range was 23–100${}^{\circ }$C, compared to 35–85${}^{\circ }$C. The larger temperature range required additional time to heat the TCA and allowed further contraction of the TCA. Surprisingly, the average cooling time for Phase I was smaller than the average cooling time for Phase II (11.42±1.33 s vs. 12.54±2.31 s), despite the larger temperature range (100–35${}^{\circ }$C for Phase I and 85–35${}^{\circ }$C for Phase II). This discrepancy can be accounted for by considering that different TCAs were used for the two different experiments, and each TCA had its own range of cooling times. Specifically, the average cooling times for the three TCAs used in Phase I were 12.79, 10.07, and 11.4 s, whereas the average cooling times for the four TCAs used in Phase II were 12.00, 9.91, 12.4, and 15.86 s. Since the last TCA used in Phase II has a longer cooling time than the other TCAs, the overall average cooling time for Phase II is longer than that for Phase I. The variations in TCA performance occur due to their fabrication parameters, such as the number of coils added during the twisting phase of fabrication (13,29), and these variations contribute to the discrepancies seen when comparing experimental results where different TCAs were used. Additionally, Phase I was completed in early spring, and Phase II was completed during the middle of summer, and thus the average ambient temperature and room humidity were higher.

## 5. Sources of error, study limitations, and future work

This paper presents the design of a fabric channel to assist with integrating TCAs into soft wearable robotic devices. While there are several advantages to the channel design and cooling apparatus, such as its flexibility and portability, there are a few limitations to the design that should be mentioned.

The primary disadvantage to this design is that air pumps are not silent actuators, and they produce noise during use. Their volume approaches that of conversation, which some users may find difficult to ignore. This is especially a concern for applications in stroke rehabilitation, where many patients are elderly and may be more sensitive to environmental noise. This problem could be overcome by continuing to investigate other means of circulating air. It is possible that a larger fan could overcome the flow resistance of the channel and achieve the same air flow rate with less noise. Alternatively, the pump could be surrounded by acoustic insulation such as rock wool or fiberglass.

Another means of circulating air would also be advantageous if the air pressure or air flow could be increased, without sacrificing the portability of the system. Increasing the air flow would allow the cooling time of the TCA to be further reduced, and increasing the air pressure would ensure that this design could work for longer TCAs that would require longer channels. It is uncertain whether the selected miniature air pump could accommodate longer channels, as it may not be strong enough to overcome the increased air flow resistance. Future work should investigate the relationship between the channel dimensions and air flow to aid with pump selection.

Phase I determined that the best dimensions for a channel, out of those tested, for a 4-ply TCA was 10 mm in width and 8 mm in height (10×8), which was the largest channel. It is possible that increasing the size of the channel more could further improve the performance of the TCA (i.e., reduce the cooling time); however, increasing the height beyond 8 mm would cause the system to protrude too much from the user’s limb, and increasing the width more than 10 mm would reduce the maximum number of channels that can be placed in contact with the limb when multiple TCAs are used. It is also likely that the ideal channel size will change depending on the TCA; for example, 2-ply TCAs are considerably smaller than 4-ply TCAs, and the optimal channel dimensions for them could be a smaller channel size.

There are some sources of error that were present in the Phase I and Phase II experiments. For example, it was more difficult to place the TCAs in some of the channels (especially the ones with the 4 mm height), causing additional downtime between trials. Since it is unknown how quickly the TCA behavior changes when not in use, the amount of variance for the first repetition in each channel changes. This was not a concern for Phase II, since the largest channel was used, making it easy to place the TCA in the channel.

Other sources of variation in the results come from differences in the TCAs and channels due to the fabrication procedure and placement of the TCA in the channel. It was observed that once a TCA was in a channel, the parameters were reasonably consistent. However, if the TCA was removed and put in the same channel, the values for the parameters were noticeably different. This indicated that the placement of the TCA in the channel is critical, and is a source of variation in the results. A limitation of the current channel design is that there is no means to guarantee consistent placement, however, if this design was implemented in a wearable device, the TCA would not be repeatedly removed and replaced in the channel. Future work could devise a method of ensuring the TCA is centered in the channel, such as adding a guide.

Another minor source of error stemmed from the room temperature changing over the course of data collection. The room temperature varied between 21.2–23.5${}^{\circ }$C (average of 21.8${}^{\circ }$C) throughout Phase I and 22.2–23.1${}^{\circ }$C (average of 22.6${}^{\circ }$C) during Phase II. This impacts the results as the ambient room temperature influences the temperature of the air being blown through the channel and the heat transfer rate between the TCA and the environment.

Finally, when collecting data, it was noticed that the temperature of the TCA continued to increase by 3–7${}^{\circ }$C over 0.3–0.6 s after the power to the TCA was turned off. Since this effect was not observed when the temperature of the TCA was measured using a thermal camera, a possible explanation is incomplete contact between the temperature sensor and the TCA, resulting in slower heat transfer between them and hence a delay in the temperature sensor reading. This may be rectified by ensuring complete contact between the TCA and the temperature sensor by using a thermal paste, however, future work should perform more validation on the temperature sensor.

## 6. Conclusion

This paper presented the design and fabrication procedure for fabric channels as a means of housing and cooling TCAs on wearable robotic devices. The fabric, nylon pack cloth with a TPU coating, is lightweight and easy to sew. A TCA can be placed in the channel and air can be blown over the TCA using a miniature air pump.

The primary factors in the channel design that would influence the cooling time of the TCA are the channel dimensions. Thus, an experiment was completed to determine the effect that changing the height and width of the channel had on TCA performance. Nine channel sizes were tested with heights of 4, 6, and 8 mm and widths of 6, 8, and 10 mm. Overall, the average cooling time and heating time of the TCA decreased as the channel size increased. The stroke of the TCA was not significantly changed by channel size, provided that the height of the channel was greater than 4 mm. The channels with a height of 4 mm generally performed the worst, and it is hypothesized that this is due to contact between the TCA and the channel walls due to the smaller size. The best channel was the 10×8 channel, as it had a low cooling and heating time, and a comparable stroke to other channels.

The 10×8 channel was utilized in a second experiment where the performance of the TCA in the channel was compared to the performance of the TCA outside of the channel. The best TCA performance occurred with active cooling without the channel, however that is not a practical setup for wearable devices as the exposed TCA could burn the user, get caught on an object, or become damaged. There was a significant decrease (42%) in cooling time between passive cooling without a channel and active cooling in a channel (21.71±1.24 s vs 12.54±2.31 s).

Future work should investigate means of improving the channel design, such as modifying the inlet, testing other materials, adding holes, or further increasing the dimensions. Additionally, other cross sectional shapes could be investigated with the addition of rigid supports to ensure that the channel remains open. Future work should also verify the ease of integration of the channel with a wearable device.

## Data Availability

The raw data supporting the conclusions of this article will be made available by the authors, without undue reservation.

## References

[1] Boulanger J Lindsay M Gubitz G Smith E Stotts G Foley N, et al. Canadian stroke best practice recommendations for acute stroke management: prehospital, emergency department,, acute inpatient stroke care, 6th edition, update 2018. Int J Stroke. (2018) 13:949–84. 10.1177/1747493018786616 30021503

[2] Dworzynski K Ritchie G Fenu E MacDermott K Playford ED. Rehabilitation after stroke: summary of nice guidance. BMJ. (2013) 346:f3615. 10.1136/bmj.f3615 23760965

[3] Kwakkel G van Peppen R Wagenaar RC Dauphinee SW Richards C Ashburn A, et al. Effects of augmented exercise therapy after stroke: a meta-analysis. Stroke. (2004) 35:2529–39. 10.1161/01.STR.0000143153.76460.7d 15472114

[4] Norouzi-Gheidari N Archambault PS Fung J. Effects of robot-assisted therapy on stroke rehabilitation in upper limbs: systematic review, meta-analysis of the literature. J Rehabil Res Dev. (2012) 49:479–96. 10.1682/JRRD.2010.10.0210 22773253

[5] Bertani R Melgari C Cola MCD Bramanti A Bramanti P Calabrò RS. Effects of robot-assisted upper limb rehabilitation in stroke patients: a systematic review with meta-analysis. J Neurol Sci. (2017) 38:1561–9. 10.1007/s10072-017-2995-5 28540536

[6] Maciejasz P Eschweiler J Gerlach-Hahn K Jansen-Troy A Leonhardt S. A survey on robotic devices for upper limb rehabilitation. J Neuroeng Rehabil. (2014) 11:3. 10.1186/1743-0003-11-3 24401110 PMC4029785

[7] Desplenter T Zhou Y Edmonds BP Lidka M Goldman A Trejos AL. Rehabilitative and assistive wearable mechatronic upper-limb devices: a review. J Rehabil Assist Technol Eng. (2020) 7:2055668320917870. 10.1177/2055668320917870 32435505 PMC7223206

[8] Gandolla M Antonietti A Longatelli V Pedrocchi A. The effectiveness of wearable upper limb assistive devices in degenerative neuromuscular diseases: a systematic review, meta-analysis. Front Bioeng Biotechnol. (2020) 7:450. 10.3389/fbioe.2019.00450 32039171 PMC6992540

[9] Madden J Vandesteeg N Anquetil P Madden P Takshi A Pytel R, et al. Artificial muscle technology: physical principles and naval prospects. IEEE J Ocean Eng. (2004) 29:706–28. 10.1109/JOE.2004.833135

[10] Daerden F Lefeber D. Pneumatic artificial muscles: actuators for robotics and automation. Eur J Mech Environ Eng. (2002) 47:11–21.

[11] Cappello L Galloway K Sanan S Wagner D Granberry R Engelhardt S, et al. Exploiting textile mechanical anisotropy for fabric-based pneumatic actuators. Soft Robot. (2018) 5:662–74. 10.1089/soro.2017.0076 30024312

[12] Connolly F Wagner DA Walsh CJ Bertoldi K. Sew-free anisotropic textile composites for rapid design, manufacturing of soft wearable robots. Extreme Mech Lett. (2019) 27:52–8. 10.1016/j.eml.2019.01.007

[13] Haines C Lima M Li N Spinks G Foroughi J Madden J, et al. Artificial muscles from fishing line, sewing thread. Science (New York, NY). (2014) 343:868–72. 10.1126/science.1246906 24558156

[14] Edmonds BP. *Feasibility of twisted coiled polymer actuators for use in upper limb wearable rehabilitation devices* [PhD thesis]. Western University (2020).

[15] Kianzad S. *A treatise on highly twisted artificial muscle: thermally driven shape memory alloy and coiled nylon actuators* [master’s thesis]. University of British Columbia (2015).

[16] Yip MC Niemeyer G. On the control and properties of supercoiled polymer artificial muscles. IEEE Trans Robot. (2017) 33:689–99. 10.1109/TRO.2017.2664885

[17] Edmonds BP Trejos AL. Design of an active cooling system for thermally activated soft actuators. *16th IEEE International Conference on Rehabilitation Robotics (ICORR)*; 2019 Jun 24–28; Toronto, Canada. p. 368–373. 10.1109/ICORR.2019.8779498 31374657

[18] Lizotte A Trejos AL. Evaluation of a fabric channel cooling apparatus for twisted coiled actuators. *Canadian Conference on Electrical and Computer Engineering (CCECE)*; 2022 Sep 18–20; Halifax, Canada. Accepted.

[19] Higueras-Ruiz DR Feigenbaum HP Shafer MW. Moisture’s significant impact on twisted polymer actuation. Smart Mater Struct. (2020) 29:125009. 10.1088/1361-665X/abc061

[20] [Dataset] American Academy of Audiology. Levels of noise (2012). Available at https://audiologyweb.s3.amazonaws.com /migrated/NoiseChart\_Poster-\%208.5x11.pdf\_5399b289427535.32730330.pdf (Last accessed May 23, 2021).

[21] Tsuji W Yoshida K Asahara S. The coefficients of friction of various fibers by Roeder’s method. Sen’i Gakkaishi. (1985) 41:T211–20. 10.2115/fiber.41.5/T211

[22] Lin TA Lou CW Lin JH. The effects of thermoplastic polyurethane on the structure and mechanical properties of modified polypropylene blends. Appl Sci. (2017) 7:1254. 10.3390/app7121254

[23] Greene LC Hardy JD. Adaption of thermal pain in the skin. J Appl Physiol. (1962) 7:693–6. 10.1152/jappl.1962.17.4.693 13901526

[24] Defin R Shachal-Shiffer M Hadgadg M Peretz C. Quantitative somatosensory testing of warm and heat-pain thresholds: the effect of body region and testing method. Clin J Pain. (2006) 22:130–6. 10.1097/01.ajp.0000154048.68273.d8 16428946

[25] Ertel JD Mascaro SA. Dynamic thermomechanical modeling of a wet shape memory alloy actuator. J Dyn Syst Meas Control. (2010) 132:051006. 10.1115/1.4002067

[26] Piao C Jang H Lim T Kim H Choi H Hao Y, et al. Enhanced dynamic performance of twisted and coiled soft actuators using graphene coating. Compos Part B: Eng. (2019) 178:107499. 10.1016/j.compositesb.2019.107499

[27] Incropera FP Dewitt DP Bergman TL Lavine AS. Fundamentals of heat and mass transfer. 6th ed. Hoboken, New Jersey, USA: John Wiley and Sons (2007).

[28] Urone PP Hinrichs R Dirks K Sharma M. College physics. Houston, Texas, USA: OpenStax (2012). Available at: http://cnx.org/content/col11406/latest/

[29] Cho Kh Song M Jung H Park J Moon H Koo J, et al. A robotic finger driven by twisted and coiled polymer actuator. *SPIE Smart Structures and Materials and Nondestructive Evaluation and Health Monitoring*; 2016 Mar 20–24; Las Vegas, USA. p. 97981J.

