# Supplementary material for: Active cooling of twisted coiled actuators via fabric air channels
Source: Front Rehabil Sci. 2022 Nov 30;3:1016355. doi: 10.3389/fresc.2022.1016355 (PMC9748810; doi:10.3389/fresc.2022.1016355)
Supplement: Supplementary file 1 [file Datasheet1.pdf]

Supplementary Material

1 SUPPLEMENTARY TABLES AND FIGURES

Table S1. Adjusted p values from the post-hoc testing for Phase I: Evaluating the Impact of Channel Height and Width on TCA Performance. Values below 0.001 are reported as 0 and comparisons that are not statistically significant are highlighted in red.

	Sheath	6×4	6×6	6×8	8×4	8×6	8×8	10×4	10×6	10×8
Cooling Time	6×4	—	0	0.648	0.468	0	0	0	0	0
	6×6	0	—	0	0	1	1	0.108	1	0.025
	6×8	0.648	0	—	1	0	0	0	0	0
	8×4	0.468	0	1	—	0	0	0.004	0	0
	8×6	0	1	0	0	—	1	0	1	0
	8×8	0	1	0	0	1	—	0	1	0.051
	10×4	0	0.108	0	0.004	0	0	—	0	0
	10×6	0	1	0	0	1	1	0	—	0
	10×8	0	0.025	0	0	0	0.051	0	0	—
Heating Time	6×4	—	0.424	1	1	0.004	0.002	1	0	0
	6×6	0.424	—	1	0.014	1	1	0.005	0.003	0
	6×8	1	1	—	1	0.008	0.006	1	0	0
	8×4	1	0.014	1	—	0	0	1	0	0
	8×6	0.004	1	0.008	0	—	1	0	0.284	0
	8×8	0.002	1	0.006	0	1	—	0	1	0.002
	10×4	1	0.005	1	1	0	0	—	0	0
	10×6	0	0.003	0	0	0.284	1	0	—	1
	10×8	0	0	0	0	0	0.002	0	1	—
Stroke	6×4	—	0	0	0	0	0	0	0	0
	6×6	0	—	0.974	0	0.136	0	0	0.793	0.128
	6×8	0	0.974	—	0	1	1	0	1	1
	8×4	0	0	0	—	0	0	1	0	0
	8×6	0	0.136	1	0	—	0.251	0	0.917	1
	8×8	0	0	1	0	0.251	—	0	0.068	1
	10×4	0	0	0	1	0	0	—	0	0
	10×6	0	0.793	1	0	0.917	0.068	0	—	1
	10×8	0	0.128	1	0	1	1	0	1	—

Table S2. Adjusted p values from the post-hoc testing for Phase II: Comparison of TCA Performance With and Without the Channel. Values below 0.001 are reported as 0 and comparisons that are not statistically significant are highlighted in red.

	Case	1. Sheath Active Cooling	2. No Sheath Active Cooling	3. Sheath Passive Cooling	4. No Sheath Passive Cooling
Cooling Time	1	—	0	0	0
	2	0	—	0	0
	3	0	0	—	0
	4	0	0	0	—
Heating Time	1	—	0	0	0
	2	0	—	0	0
	3	0	0	—	0.013
	4	0	0	0.013	—
Stroke	1	—	0	0	0
	2	0	—	0	0
	3	0	0	—	0.004
	4	0	0	0.004	—
Hysteresis	1	—	1	0.120	0.180
	2	1	—	0.012	0.048
	3	0.120	0.012	—	0.648
	4	0.180	0.048	0.648	—
